# A fast multispectral light synthesiser based on LEDs and a diffraction grating

**DOI:** 10.1038/srep32012

**Published:** 2016-08-25

**Authors:** Gregor Belušič, Marko Ilić, Andrej Meglič, Primož Pirih

**Affiliations:** 1 Department of Biology, Biotechnical Faculty, University of Ljubljana, Ljubljana, Slovenia; 2 Sokendai (The Graduate University of Advanced Studies), Hayama, Kanagawa, Japan

## Abstract

Optical experiments often require fast-switching light sources with adjustable bandwidths and intensities. We constructed a wavelength combiner based on a reflective planar diffraction grating and light emitting diodes with emission peaks from 350 to 630 nm that were positioned at the angles corresponding to the first diffraction order of the reversed beam. The combined output beam was launched into a fibre. The spacing between 22 equally wide spectral bands was about 15 nm. The time resolution of the pulse-width modulation drivers was 1 ms. The source was validated with a fast intracellular measurement of the spectral sensitivity of blowfly photoreceptors. In hyperspectral imaging of *Xenopus* skin circulation, the wavelength resolution was adequate to resolve haemoglobin absorption spectra. The device contains no moving parts, has low stray light and is intrinsically capable of multi-band output. Possible applications include visual physiology, biomedical optics, microscopy and spectroscopy.

Many optical experiments require a near-ultraviolet and visible light source with adjustable wavelength and intensity. A classical single-band light source used in e.g. visual physiology experiments consists of a Xenon arc lamp (XBO), a shutter, a graded neutral density wedge and a monochromator or a filter wheel^1^,^2^. Usually, the bandwidth of the passband is 5~20 nm (full width at half maximum, FWHM), and the wavelength and intensity changes can be achieved within about a second. In order to achieve a multi-band output and faster changes several beams have to be combined via beam-splitters. Hyperspectral imaging systems^3^,^4^ either use a hyperspectral camera and a white light source^5^,^6^, or a monochrome camera and a hyperspectral source^7^,^8^.

An alternative to a monochromator-based system is a multi-band stimulator based on light emitting diodes (LEDs). Light uniformity can be achieved with an integrating sphere, a light guide with high numerical aperture or a diffuser^9^,^10^,^11^,^12^. LEDs have typical bandwidths (FWHM) of 10~50 nm, and especially the UV LEDs have tails in the visible spectrum^13^, so additional spectral filters may be needed if narrower spectral bands are required.

The light flux cannot be concentrated more than its source radiance due to conservation of *étendue*, the product of the source area and its radiant solid angle. Since the LEDs cannot be in the same location, these sources either suffer from low spatial uniformity or from large *étendue*, limiting their usability especially for microscopy. It is possible to combine beams of different wavelengths without *étendue* increase either by using dichroic beamsplitters or by employing the dispersion innate to refractive or diffractive optical elements. Wavelength combining by prisms was for the first time put forward by Newton, who proposed in his First Book of Opticks^14^ to synthesise white light “*By mixing colour’d Lights to compound a beam of Light of the same Colour and Nature with a beam of the Sun’s direct Light […]*”. More recently, gratings are being routinely used in laser optics for wavelength combining^15^,^16^,^17^.

Here, we present a light source consisting of a series of LEDs, a planar reflective diffraction grating acting as a wavelength combiner, a launching lens and a light guide. The source is intrinsically multi-band, contains no moving parts, and is small and inexpensive in comparison with XBO-based sources. The multi-band light synthesiser is suitable for applications that require a high time resolution, e.g. time-resolved spectroscopy, or a small *étendue*, e.g. in microscopy.

## Results

### The optical setup

A reflective planar grating with groove density *G* (e.g. 1200~2400 grooves/mm) diffracts an input beam of wavelength *λ*, incident under angle *α* to the grating normal, into several diffracted orders *m*, exiting under angles *β*_*m*_(*λ*), according to the grating equation (sin*α* + sin*β*_*m*_ = *mGλ*)^18^. In a classical spectrograph, the input angle *α* is kept constant and the detector spans a range of first order exit angles *β*_*+*1_(*λ*). In the wavelength combiner presented here, the input beams span a range of angles *α*(*λ*), while the first order exit angle *β*_*+*1_ is constant.

**Figure 1:**
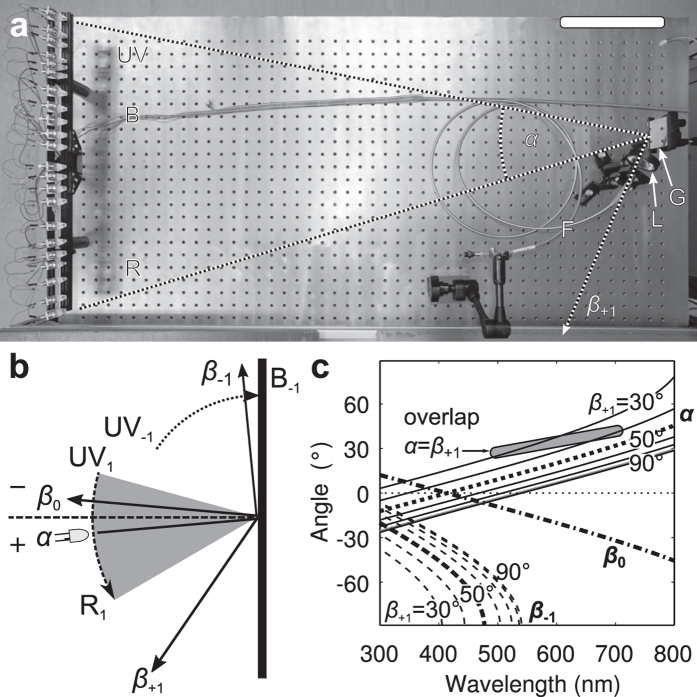
The optical table layout and principle. (**a**) Top-view of the optical bench. The LEDs with collimators are mounted on a profile (left side). G: Grating; L: Launching lens, F: fibre; scale bar: 20 cm. (**b**) The principle of wavelength combining with a grating under a constant exit angle. An LED, incident angle *α*, produces a specular reflection at the angle *β*_0_ = −*α*, and a positive first order diffraction at the angle *β*_+1_. The negative first order angle *β*_−1_ may exist for shorter wavelengths. The dashed arc represents the approximate input angle range for wavelengths 300 to 700 nm for grating density *G* = 1850 mm^−1^, and the dotted arc indicates the negative first order beams (UV to blue). UV_*m*_, B_*m*_, R_*m*_ indicate the positions of diffracted orders *m*, (**c**) Input angles for a grating with *G* = 1850 mm^−1^, for exit angles *β*_+1_ between 30° and 90°. Solid lines represent input angles *α*. Dashed lines represent negative order angles *β*_−1_. Thick lines represent the configuration with exit angle *β*_+1_ = 50°, used in the prototype. Input angle *α* (dotted), specular reflection *β*_0_ (dot-dashed) and negative first order *β*_−1_ (dashed). Shaded area shows the design constraint for small exit angles (*β*_+1_ <50°), where the input angles may overlap with the exit angle (*α* = *β*_+1_) in the wavelength range 500 nm to 700 nm.

Figure 1 shows the prototype built around a ruled reflective diffraction grating with groove density *G* = 1850 mm^−1^ and a row of LEDs (Fig. 1a). The input angles subtend about 30° and the first order beam exit angle is set to a constant *β*_+1_ = 50° (Fig. 1b). The combined beam is launched to a light guide with a single aspheric lens (Fig. 1a). All inputs are also diffracted to the zero order, while the inputs with wavelengths below ~500 nm are additionally diffracted to the negative first order *β*_−1_ (Fig. 1b,c).

**Figure 2:**
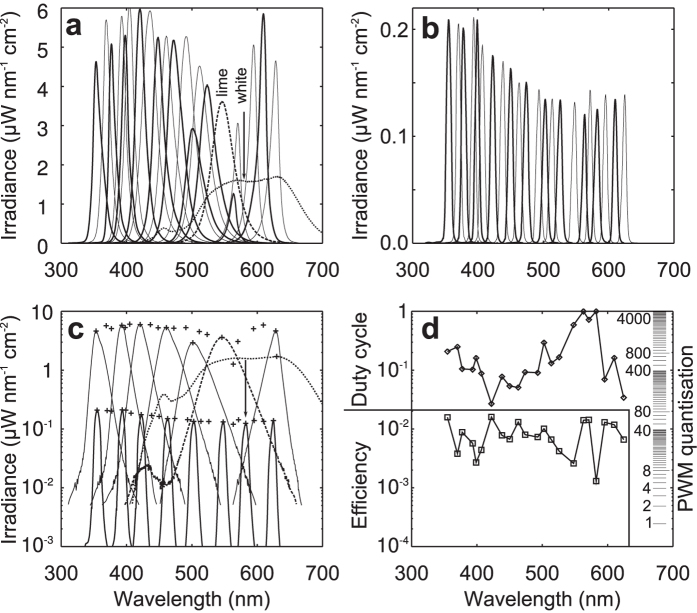
Bandwidths and efficiency of the wavelength combiner. (**a**) Spectral irradiances measured at the tip of the LEDs. Dotted and dashed line represent the white and green phosphorescent LEDs, respectively. (**b**) Spectral irradiances of the individual LEDs at the fibre output, adjusted for equal photon flux via PWM. Thick and thin lines show the spectra of consecutive LEDs. (**c**) The data of panels (**a**,**b**) compared in a semi-logarithmic plot. The dotted and dashed lines represent the irradiance spectra of a white LED and green phosphorescent LED, respectively. The arrow shows the spectral narrowing of the white LED. The crosses mark peak irradiances of the LEDs. (**d**) A semi-logarithmic plot of the overall coupling efficiency of individual LEDs (squares), range 0.1~1.6%, and the PWM duty cycle used to achieve equal photon fluxes at the fibre output (2% for LED 420 nm, 100% for the white LED) (diamonds). The scale on the right side shows the PWM quantisation levels.

Figure 2 shows the measured bandwidth and efficiency of the prototype. The bandwidths of the installed LEDs vary substantially (Fig. 2a). However, the combination of grating and fibre strongly narrows and unifies the bandwidths (FWHM = 6–8 nm, Fig. 2b) and the individual output beams have little stray light (Fig. 2b,c). Using commercially available quasi-monochromatic LEDs, we could cover the wavelength range 350–630 nm in approximately equally spaced intervals, except for the regions 520–563 and 570–594 nm. We filled these gaps with phosphorus-based lime green and white LEDs (Fig. 2a,c, dotted and dashed lines).

Due to the restricted aperture of the collecting fibre, only part of the spectral band of each LED is captured. The coupling efficiency of the LEDs (the ratio of the irradiance at the fibre output and the LED tip) is further reduced due to vignetting loss, to 0.1%~1.6% (Fig. 2d). Expectedly, the coupling efficiency of the white LED was the lowest (Fig. 2c arrow, Fig. 2d). To achieve isoquantal (i.e. equal photon flux) output on all channels, the LEDs with the highest coupling efficiency were attenuated to about 2% (Fig. 2d).

The output of the LEDs was fully linear with the pulse width modulation (PWM) duty cycle and showed good temporal stability over the whole intensity range. The PWM driver has 1 kHz update frequency and 12-bit resolution, which allows for a 3.6 decade dynamic range. The remaining quantisation reserve (Fig. 2d, right scale) allows for at least one decade of general attenuation without serious quantisation errors.

### Intracellular spectral sensitivity measurements

**Figure 3:**
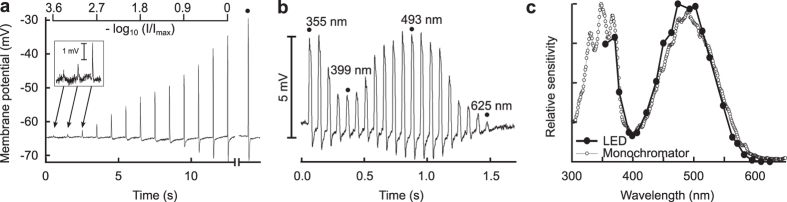
Intracellular spectral sensitivity measurements from R1-6 photoreceptors of a blowfly. (**a**) Membrane potential trace of an R1-6 blowfly photoreceptor upon stimulation with a graded series of 25 ms pulses with 474 nm light. The pulse intensity was graded in a geometric series (PWM 1, 2, 4, …, 2^12^). The decadic logarithm of the light intensity is indicated on the top axis. The last response (dot) was evoked with a 350 mA pulse from a power LED peaking at 525 nm, directed into the zero-order beam. (**b**) Membrane potential trace of an R1-6 blowfly photoreceptor upon isoquantal spectral stimulation with 25 ms pulses from the LED synthesiser. (**c**) Spectral sensitivity obtained from an average of ten sweeps with the LED synthesiser (solid circles). For comparison, the spectral sensitivity obtained from a single spectral sweep with a classical photostimulator is shown (open circles).

We used the LED synthesiser to measure the spectral sensitivities of R1–6 photoreceptors of the blowfly *Calliphora vicina* (white-eyed mutant Chalky) by intracellular recordings (Fig. 3), applying 25 ms monochromatic light pulses. In the calibration run, applying 474 nm light, we used a geometric series of intensities, corresponding to 0.3 decade steps spanning 3.6 decades. The maximal response amplitude was about 35 mV. The flash from a high-current green LED (525 nm, 350 mA) launched through the zero order input angle produced a non-saturating response with 40 mV amplitude (Fig. 3a). To reach saturation, the LED output would need to be about one decade stronger.

An isoquantal spectral run at 22 wavelengths could be obtained in less than two seconds (Fig. 3b). Due to the short duration of the sweep, we were able to repeat ten measurements within 30 seconds. The spectral sensitivity curve of Fig. 3c was obtained by averaging the response amplitudes and transforming them with the inverse function of the Hill (Naka-Rushton) sigmoid fitted to the amplitudes of the calibration sweep. As a control, we performed the same spectral sensitivity measurement using a classical photostimulator, yielding very similar results (Fig. 3c).

### Hyperspectral imaging of blood oxygenation

**Figure 4:**
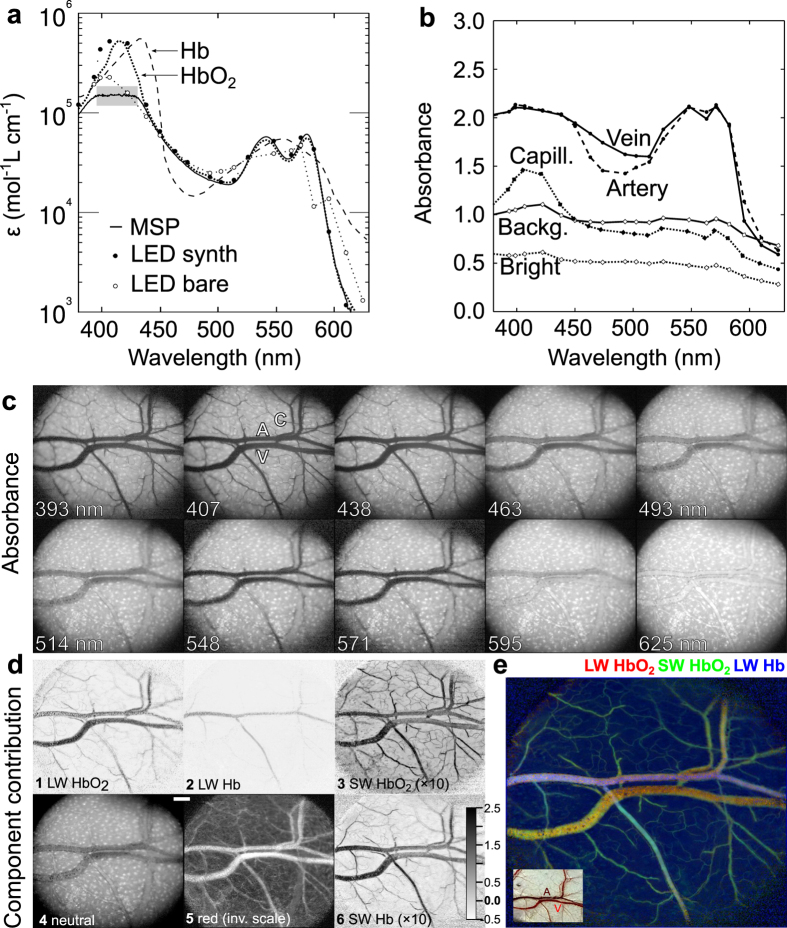
Imaging spectroscopy of lysed human blood and of *Xenopus* skin *in situ*. (**a**) Comparison of tabulated molar extinction spectra of human oxyhaemoglobin (HbO_2_, dotted line) and deoxyhaemoglobin (Hb, dashed line) with scaled absorbance measurements of diluted fresh human blood. The spectrophotometric measurement (solid line) closely follows the HbO_2_ spectrum above 440 nm; at wavelengths below 440 nm, the measurements are unreliable (shaded area). The absorbance spectrum obtained with imaging spectroscopy using the LED synthesiser (solid circles) closely follows the HbO_2_ spectrum above 420 nm. The absorbance spectrum obtained with bare LEDs (open circles) fails to reproduce the fine structure of the HbO_2_ absorbance spectrum at 530–580 nm. (**b**) Absorbance spectra from the artery (A), vein (V), capillary (C), skin background and bright spots. (**c**) Images of the frog skin obtained with the LED synthesiser. The thin capillaries are best seen in the images between 390 and 430 nm. At 625 nm, the thick vessels show a bright lumen and are surrounded by dark pigmented cells. (**d**) Images obtained by unmixing with six absorbance components: HbO_2_ and Hb above (1, 2) and below 455 nm (3, 6), a spectrally neutral (4) and a red component (5). The LW Hb component discriminates the vein from the artery. The SW Hb component shows an image of the capillaries. (**e**) False-color image of frog skin. Components 1–3 from (**d**) were allocated to RGB channels (Red, LW HbO2; Green, SW Hb; Blue: LW Hb). Inset: colour photo of the analysed patch. Scale bar in (**d**): 1 mm.

Due to its small *étendue*, the LED synthesiser is a highly suitable source for hyperspectral microscopic imaging (Fig. 4). In order to test the wavelength resolution of the system, we first acquired an image stack of fresh human blood lysed in distilled water, using the LED synthesiser and a monochrome CMOS camera coupled to a microscope. The blood extinction spectrum obtained from the hyperspectral stack was in close agreement with that measured with a spectrophotometer, and with the tabulated extinction spectrum of human oxyhaemoglobin^19^. We also measured the blood spectrum using bare LEDs (Fig. 2a,c), but this measurement failed to show the fine structure of the oxyhaemoglobin (Fig. 4a), thus demonstrating the advantage of bandwidth narrowing by the grating and the fibre.

To investigate the blood absorption spectra *in situ*, we performed hyperspectral imaging of *Xenopus* abdominal skin, a respiratory tissue where the veins contain oxygenated blood and the arteries contain deoxygenated blood^20^. We illuminated the skin from the outside and acquired an image stack of the internal side of the skin (Fig. 4b). Figure 4c shows the absorbance spectra of an artery, a vein, a capillary and adjacent tissue. We processed the image pixels with a linear unmixing procedure^21^,^22^, using six spectral components. The oxyhaemoglobin (HbO_2_) and deoxyhaemoglobin (Hb) extinction spectra^19^ were cut into two long wavelength (LW) and two short wavelength (SW) components, and additionally we used a spectrally neutral and a red component.

The component contributions are presented in Fig. 4d. The LW oxyhaemoglobin component (Fig. 4d1) reveals both arteries and veins, while the LW deoxyhaemoglobin component shows the arteries only (Fig. 4d2). The two SW components (Fig. 4d3,d5) emphasize the thin capillaries. The neutral component (Fig. 4d4) shows melanocytes lining the vessels and the mucous glands as dark and bright spots, respectively. The red component, showing thicker vessels, was necessary to account for the reduced absorbance above 580 nm, which is presumably due to scattering by erythrocytes (Fig. 4d5).

## Discussion

We constructed an LED synthesiser spanning the wavelength range 350–630 nm. With the prototype, based on inexpensive low-current LEDs, we were able to successfully perform physiological and spectroscopic experiments that would otherwise require a much more complex and expensive light source. The heart of the system is a planar reflective diffraction grating, which both combines and cleans (i.e. narrows the bandwidth of) the light inputs. The main optical components are 5 mm epoxy-mould LEDs, a grating, an aspheric lens and a light guide. The custom-made parts are the LED holders with collimator lenses. The size of the holders and the grating density determine the optical bench length, currently about 1 m. The electronics consists of an Arduino Due microcontroller and a 12-bit PWM driver. The overall cost of the components is about €700 (€100 for the LEDs, €100 for the electronics, €500 for the optical components without the breadboard).

Compared with systems based upon a white light source and a monochromator, the LED synthesiser has a strongly reduced stray light component, because the LED channels are switched on only when their output is needed. Moreover, while the crosstalk from the second diffraction order (e.g. UV crosstalk in the range 580–800 nm) is problematic with XBO-monochromator sources, this is not an issue in the case of LEDs, because they have little output at half the main peak wavelength.

We have tested the capability of the prototype in a fast measurement of the spectral sensitivity of blowfly photoreceptors by intracellular recording. The LEDs were attenuated to be isoquantal *via* PWM. A single spectral run, obtained within seconds, was sufficient for measuring an accurate sensitivity spectrum. The responses of blowfly photoreceptors to maximal intensity pulses were non-saturating, but this is not a serious limitation, since reaching saturation is not a necessity for many measurement paradigms. Also, because the 1 kHz switching frequency is sufficiently above the 60 Hz corner frequency of the blowfly R1–6 photoreceptors^23^, the measured responses did not show significant ripple. The LED wavelength spacing of about 15 nm is adequate for photoreceptor measurements, with the rare exceptions of the spectral sensitivity of fly photoreceptors in the UV^24^ or of the narrow-peaked red photoreceptors of some butterflies^25^.

Several measurement paradigms for visual physiology could be implemented autonomously in the microcontroller: selective adaptation^26^, lock-in signal extraction^27^, various versions of voltage clamp by light^28^,^29^,^30^, or random spectral stimulation with maximal length binary sequence (m-sequence) stimulation^31^ in the wavelength domain. The LEDs could also be connected to analogue LED drivers if white-noise stimulation^10^,^32^ is desired. Another attractive option for wide field stimulation^33^ would be to couple the LED synthesiser to a digital mirror device (DMD) projector. The effective intensity of the LED synthesiser coupled to a DMD projector could be increased using the extended Maxwellian view^34^.

We have used the LED synthesiser as a light source for microscopic hyperspectral imaging of blood oxygenation. A simple unmixing procedure with six components revealed arteries, veins and capillaries. Absolute haemoglobin concentrations and tissue oxygen saturation levels can be estimated with the unmixing, provided that sufficiently rigorous calibration is performed. For oxymetry of the retinal fundus, the LED synthesiser and a monochrome camera could be easily coupled to an ophthalmoscope^7^.

When using white light illumination for spectrophotometric absorbance measurements, stray light and the limited dynamic range of diode-array spectrophotometers often limit the measurable absorbance range to about three decades (Fig. 4a). Although this dynamic range can be expanded by using a filter that selectively blocks the light in the wavelength range where the sample has low absorbance, the LED synthesiser offers the same functionality, but with added flexibility: the spectrometer reading can be optimized by dynamically adjusting the source composition *via* a similar procedure as being used for component unmixing. Due to high linearity and stability of the LEDs, repeated reference measurements are not required. As the LEDs have temporal bandwidths in excess of 1 MHz, the LED synthesiser can be used as the source for high speed spectroscopic measurements^35^ by employing faster driver electronics.

In colour science, the LED synthesiser can be used as the light source for BRDF (bidirectional reflectance distribution) measurements with an imaging hemispherical scatterometer^36^, or with an imaging angular reflectometer^37^. If larger scenes need to be illuminated from several angles, the LEDs and output fibres can be stacked in vertical columns around a single grating, or several LED synthesisers can be operated in synchrony.

The multi-band light synthesis by means of wavelength combining presented here brings about important advantages over solutions with broad-band light sources and dichroic mirrors or monochromators. The reflective diffraction grating transforms the often complex spectra of the various LEDs into bands with uniform bandwidths. Furthermore, the LEDs have a long operational life and the optical system has no moving parts. The LED synthesiser has adequate light output for transmission and reflection light microscopy, and it may become a standard tool for visual physiologists and a rig in advanced neurophysiology lab courses^38^. Particularly appealing are possible applications in biomedical imaging and in clinical electrophysiology of vision. With brighter LEDs, the synthesiser will become also attractive for fluorescence microscopy, optogenetics, and as a general hyperspectral illumination source.

## Methods section

### Theory of wavelength combining

A grating with groove density *G* diffracts a light beam of wavelength *λ*, incident at an angle *α* to the grating normal, into diffraction orders (*m* = … −1, 0, 1, …), exiting at angles *β*_*m*_, according to the grating equation^18^:


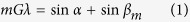


The zero order (*m* = 0) represents specular reflection: *α* = −*β*_0_. Non-zero orders are diffracted when |*mGλ*| < 2 and are evanescent otherwise. Solving Eq. 1 for the limiting condition (grazing exit angle, sin *β*_+1_ = 1) yields the relation between the grating density and the highest passable wavelength: *λ*_+1_ = 2/*G*. For the UV-visible range (300–700 nm) it follows that *G* < 2700 mm^−1^.

The light synthesiser works as a beam combiner. The beams are incident upon the grating at input angles *α*(*λ*) and exit under a constant exit angle of the positive first order diffraction, *β*_*+*1_:


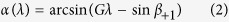


A design constraint of the wavelength combiner is that the exit angle *β*_+1_ should not overlap with the input angles. The overlap is avoided when:


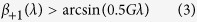


For a grating with *G* = 1850 mm^−1^ (*λ*_+1_ = 1081 nm), the exit angle *β*_+1_ should be larger than 47° to avoid the overlap below *λ* = 800 nm. Another point of consideration is the existence of the negative first order. The grating equation (Eq. 1) yields as the limiting case for orders *m* = (+1, −1):


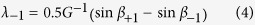


The wavelength limit for which the negative order exists for the particular design *λ*_−1_(*G*, *β*_+1_) is found by setting sin *β*_−1_ = −1. By further setting the exit angle to *β*_+1_ = 90° (sin *β*_+1_ = 1), the maximal wavelength of the negative order is *λ*_−1, max_ = *G*^−1^. For the grating density 1850 mm^−1^, the maximal negative first order wavelength is 540 nm.

### Optical setup

We used a blazed grating with density *G* = 1850 mm^−1^ (Thorlabs GR50-1850, nominal blaze wavelength 500 nm). The exit angle was set to *β*_+1_ = 50° (Fig. 1c). The exit beam was launched with an aspheric lens (*f* = 32 mm, ACL50832U, Thorlabs) onto an optical fibre, either an 0.4 NA, high OH, 1 mm fibre (M35L01, Thorlabs) or an 0.22 NA, fused silica, 200 μm fibre (P200-2-UV-VIS, Ocean Optics).

The span of the input angles for the wavelength range 350 to 630 nm was ~30°. Because the illumination cones of the LEDs were larger than the grating, the projection efficiency was approximately proportional to the projection of the input angle, cos *α*(*λ*), i.e. it is half-maximal at 60° incidence. For *G* = 1850 mm^−1^ and *β*_+1_ = 50°, the projection efficiency is down to 0.8 (80%) at 700 nm. The negative first order existed up to *λ*_*−1*_ = 472 nm (Eq. 4).

### LEDs

LEDs (epoxy mould, diameter 5 mm, maximal current 20 mA) with peak wavelengths between 350 nm and 630 nm were purchased from an on-line reseller (www.roithner-laser.com). The LEDs have been selected for small emission angles (Δ*ρ*_1/2_ = 8–15°). The spectral range was covered in steps of approximately 15 nm. 22 LEDs were mounted into aluminium T-shaped profiles (30 × 30 × 13 mm, thickness 3 mm) with collimating lenses (LT-0454, www.led-tech.de). The LEDs were secured with M3 screws along a 15 × 15 mm aluminium beam with T-slots (Open beam, www.openbeamusa.com) and mounted on the holders about a meter away from the grating (Fig. 1a). The input and output peak wavelengths differed maximally ~3 nm.

### Electronics

An Arduino Due microcontroller with ARM Cortex M3 processor (www.arduino.cc) controlled a TLC5947 chip-based 24-channel PWM LED driver (12-bit resolution, 4 MHz clock, update frequency 1 kHz, www.adafruit.com) via the SPI bus. The software that controlled the driver and communicated with the computer over the USB was written in the Arduino IDE environment, using the provided libraries.

### Calibration procedure

For calibration measurements, we used a radiometrically calibrated spectrophotometer (Flame, Ocean Optics, USA) and a photodiode with a linear amplifier (OPT-101, Texas Instruments, USA). When necessary, we used the same fibre as the one for stimulation and a collimator (74-UV, Ocean Optics, USA). The positions for mounting individual LEDs were first empirically determined by illuminating the diffraction grating with white light from the XBO lamp in reverse, via the optical fibre and the launching lens. The diffracted light was projected onto the mounting beam and the approximate positions corresponding to LED emission peaks were determined with the spectrophotometer.

To monitor the LED light, the XBO lamp was substituted with the spectrophotometer, and the LEDs were precisely positioned so that their light output around the declared emission peak was maximised. Most of the LEDs were additionally collimated with the LED holder’s collimating lens. For some LEDs, the collimating lens degraded the light output and was removed.

Light fluxes of the LEDs in the array were measured with the spectrophotometer. The efficiency of individual LEDs was calculated by dividing the output intensity from the optical fibre with the intensity measured by collecting the light with the same fibre, equipped with a collimating lens, positioned next to the LED on its optical axis.

We integrated the photon flux of each channel in a 20 nm bin around the emission peak, using the radiometrically calibrated spectrometer, which measured the light reflected from a magnesium oxide block positioned 15 cm in front of the fibre. In order to generate isoquantal stimuli, we adjusted the PWM for each channel until the irradiance integral was inversely proportional to the wavelength. Consequently, the LEDs with narrower bandwidths had somewhat higher peaks. The most effective LED had to be attenuated to a 2% duty cycle and the white LED was running at the full duty cycle (Fig. 2d).

### Classical photostimulator

The classical photostimulator was constructed from a stabilised XBO lamp (66475-150XV-R22, Newport USA), a monochromator (model 77250, Oriel/Newport) and a motorised, graded neutral density filter on a fused silica substrate (NDL-25C-4, Thorlabs). We operated the photostimulator in the wavelength range 300 nm to 700 nm with 1 nm or 5 nm steps in isoquantal mode.

### Electrophysiology

The white-eyed blowflies *Calliphora vicina* Chalky were reared in a lab culture. Experimental animals were immobilised and put into a motorised goniometric positioner stage (Standa, Lithuania), which directed a collimated fibre output towards the centre of rotation of the goniometer. The photoreceptor cells were impaled with a sharp borosilicate microelectrode (resistance ~80 MΩ) and illuminated with a series of isoquantal flashes of 25 ms duration, which were produced either with the LED synthesiser or the classical photostimulator. The photoreceptor membrane voltage was amplified with a high impedance amplifier (SEC10LX, NPI electronic, Germany). The signals were acquired using a 16-bit laboratory interface (1401micro Mk2 with Signal2 software, Cambridge Electronic Design, UK) and Stratchlyde WinWCP software^39^,^40^.

The stimulus-response relation was estimated from the calibration run (Fig. 3a) by fitting the response amplitudes *V*(*I*) to the Hill (Naka-Rushton) function:


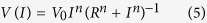


here *I* is the light intensity, *V*_0_ the maximal response, *R* the intensity needed for a half-maximal response, and *n* the slope of the sigmoid. Figure 3b presents the raw responses to the isoquantal stimuli. The intensity *I*_c_(*λ*) necessary to create a criterion response *V*_c_ then is:


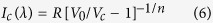


The spectral sensitivity is subsequently calculated as the normalised inverse criterion intensity^25^,^41^,^42^.

### Hyperspectral imaging

We performed spectroscopy of a human blood sample. The sample (200 μl) was collected from a finger and lysed in 5 ml distilled water. The absorbance spectra of the diluted blood were acquired on a Leitz Orthoplan microscope in transmission mode with a Flame-S spectrophotometer (Ocean Optics, USA), using the XBO source. A hyperspectral image stack was taken with a monochrome CMOS camera (Grasshopper 2.3 MP USB3, Point Grey Research, Canada), using the light output of the LED synthesiser.

We also performed hyperspectral imaging of the skin of adult African clawed frogs, *Xenopus laevis*, obtained from a commercial supplier (Xenopus Express, France). The frog was euthanised by brain concussion and the brain and spinal cord were destroyed with a steel probe inserted through the palate. The experiment was performed in a cadaver with a preserved functioning circulatory system. A euthanised frog does not breathe with its lungs, so the skin is its only functional respiratory surface. Therefore, the skin arteries carry dark red deoxygenated blood, while the veins carry light red oxygenated blood. The identity of both types of blood vessels can be determined with the application of norepinephrine, which constricts the arteries, but not the veins^20^.

An incision was made on the abdomen, exposing the inner surface of the abdominal skin of about 2 by 3 cm. The skin’s outer surface was illuminated with the LED synthesiser through a 200 μm diameter light fibre focussed with a fused silica collimator from about 2 cm distance from the skin. A monochrome camera was attached to a stereomicroscope. The individual LED stimuli were set to maximal output to obtain a hyperspectral image stack. Due to the small aperture of the stereomicroscope objective and scattering loss, skin images had to be taken with a relatively long exposure of 1 s and gain 10 dB. The images of the reference measurement were taken with 5 ms exposure and 0 dB gain.

The stacks with 20 images were preprocessed with Fiji (www.fiji.sc). Hot pixels were replaced with the median value of neighbouring pixels. The images with low signal amplitudes (UV and green wavelength ranges) were additionally filtered with a Gaussian low pass filter. The stacks were scaled down to images with 576 × 512 pixels, exported to Octave (www.octave.org) and transformed to decadic absorbance, using a reference stack. The pixel absorbance spectra were unmixed^21^ as the sum of six component spectra (short and long wavelength parts of haemoglobin and deoxyhaemoglobin extinction spectra, cut at 455 nm; neutral component and red component). The unmixing procedure was optimised to a single matrix-vector multiplication per pixel and fully vectorised to reduce the stack processing time in Octave to ~0.1 s. A detailed description of the analysis will be published elsewhere.

### Animal experiment clearance

Experiments in euthanized animals were performed at the Department of Biology of the University of Ljubljana in accordance with the national guidelines (ZZZiv-UPB 23, Ur. list RS 38/13) and approved by the Administration of the Republic of Slovenia for Food Safety, Veterinary Sector and Plant Protection (Permission no. U34401-32/2014/4).

## Additional Information

**How to cite this article**: Belušič, G. *et al*. A fast multispectral light synthesiser based on LEDs and a diffraction grating. *Sci. Rep.* **6**, 32012; doi: 10.1038/srep32012 (2016).
